# Mathematical modelling of the heterogeneity of disease progression and treatment outcomes in patients with COVID-19

**DOI:** 10.3389/fmicb.2025.1551320

**Published:** 2025-07-28

**Authors:** Guozhi Yu, Houhui Huang

**Affiliations:** ^1^Department of Bioengineering and Applied Biology, College of Life Sciences, Sichuan Agricultural University, Ya’an, China; ^2^Department of Mathematics, College of Sciences, Sichuan Agricultural University, Ya’an, China

**Keywords:** COVID-19, SARS-CoV-2, infection heterogeneity, disease progression, mechanistic model, clinical intervention

## Abstract

Pneumonia caused by SARS-CoV-2 infection is a self-limiting disease. Its progression and prognosis are highly heterogeneous among people of different ages, genders, and living with different life styles. Such heterogeneity also exists in treatment outcomes of different patients. Various physiological and pathological factors, such as renewal of pulmonary cell, number of entry receptor and viral replication, have been identified linking to the development of the disease. However, it is still unclear how these factors collectively establish a causal relationship in the course of disease progression. In this study, we built a mechanistic model to explain the dynamics of infection and progression of COVID-19. We modeled how the interaction of pulmonary cells determine the dynamics of disease progression by characterizing the temporal dynamics of viral load, infected and health alveolar cells, and dysfunctional alveolar cells. The viral and cellular dynamics captured different stages of clinical manifestations in individual patient during disease progression: the incubation period, mild symptom period, and severe period. We further simulated clinical interference at different stages of disease progression. The results showed that some medical interventions show no improvement either in reducing the recovery rate or shortening the recovery time. Our theoretical framework may provide a mechanistic explanation at the systems level for the progression and prognosis of COVID-19 as well as other similar respiratory tract diseases.

## Introduction

SARS-CoV-2 (COVID-19) is a self-limiting disease, but it can sometime develop into conditions in which patient suffer from sever acute respiratory syndrome (SARS) or acute respiratory distress syndrome (ARDS). One of the key issues remaining elusive is the heterogeneity of disease progression. Firstly, it is still not clear how the disease suddenly progresses from mild to severe condition and what dynamic mechanisms contribute to that rapid transition. For example, after several days of mild symptoms, patient’s health condition can rapidly deteriorate within 1 or 2 days. Patients with severe cases need extra mechanical ventilation or even extracorporeal membrane oxygenation (Zhao, 2003; Yang et al., 2020; Peiris et al., 2003). This has been associated with the accumulation of cytokines and cytotoxic lymphocytes, which cause massive pulmonary damage. In particular, the disease severity is attributed to the lymphocytes that are cross-reactive to the SARS-CoV-2 that causes seasonal flue (Mateus et al., 2020). In addition, disease progression showed distinct developmental patterns in people of different age groups (Guan et al., 2020), life-style (e.g. smokers vs. nonsmokers) (Smith et al., 2020; Liang et al., 2020) and biological sex (Cai, 2020; Reddy et al., 2021; Scully et al., 2020). For example, in the early stages of the pandemic, the majority of infected children and young adults developed no symptoms or mild symptoms (Dong et al., 2020; Zheng F. et al., 2020). The clinical manifestations of infections are less severe and recover shortly without medical intervention. However, infections in older groups (usually older than 60) are more likely to develop sever and critical illnesses. Epidemiological surveys across countries show worse infection outcomes in males compared to females. Studies show that many factors, including receptor density, viral load, and the accumulation of interferon, are related to the development of the disease. For example, disease progression is associated with a low abundance of lymphocytes, typically CD4^+^ and CD8^+^ cells (Diao et al., 2020; Tan et al., 2020; Zheng M. et al., 2020). The causal connection between these factors and disease progress is still unclear. Although many physiological and serological indicators are associated with the severity of this disease, it is difficult to predict on an individual level whether and when a mild case will transit into severe or critical condition. We believe that it is important and necessary to establish a quantitative framework to explain the heterogeneity of infection outcomes and disease progression in COVID-19.

Accumulating evidence shows that the excessive immune response following by viral infection is the cause of pathogenesis in COVID-19, especially in critically ill patients. Although viral load is associated with severity, significantly reduced viral load is observed in critically ill patients or during the severe phase of disease progression (Lee et al., 2015), This trend was also observed in other viral infection, including human influenza A (H5N1) (de Jong et al., 2006; Yu et al., 2011) and SARS-COV (Peiris et al., 2003). For example, type III interferons in lower respiratory tract induce barrier damage and cause susceptibility to lethal bacterial superinfections (Broggi et al., 2020). In patients at different stages of COVID-19, cytokine and chemokine profiling in peripheral blood revealed that the pathogenesis is associated with several markers of endothelial injury and thrombosis, such as IL-6 and GM-CSF (Thwaites et al., 2021). High level of cytokines are also present in severely ill patients with many other viral disease (Peiris et al., 2003; de Jong et al., 2006; Herold et al., 2008; Mehta et al., 2020; Lee et al., 2020). Inhibition of cytokines in clinical treatment significantly prevents disease progressing into critical conditions (Stone et al., 2020). In addition, some types of T cells many play an important role in the deterioration of the diseases, in particular, those that are cross-reactive among many other COVID-19 with high levels of cytotoxicity (Mateus et al., 2020; Bacher et al., 2020; Peng et al., 2020).

Various treatment strategies have been implemented to treat COVID-19 during the pandemic, resulting in remarkably varied outcomes. For example, treatment with protease inhibitors lopinavir and ritonavir showed no benefit compared to standard care in severely ill patients (Cao et al., 2020). Meanwhile, a traditional Chinese medicine formula, *Lianhuaqingwen,* showed significant antiviral capacity *in vitro*, improved the recovery rate, and shortened the treatment time of mild cases. However, the formula had no effect on severe cases (Hu et al., 2021; Runfeng et al., 2020). Many other antiviral treatments also revealed similar patterns of treatment both *in vivo* and in vitro (Pruijssers et al., 2020; Beigel et al., 2020; Wang et al., 2020). This indicates that the treatment outcome may depend on therapeutic window. Theoretical analysis showed that treatment aimed at inhibiting viral replication should be administered as early as possible (Gonçalves et al., 2020; Dodds et al., 2021), which has been confirmed by subsequent clinical trials (Hu et al., 2021; Garibaldi et al., 2021; Hung et al., 2020). Virus neutralizing treatment with convalescent plasma showed no benefit in a randomized trail (Simonovich et al., 2021), but a latter trail revealed that early administration with high titter resulted in better outcomes (Libster et al., 2021). The underlining mechanism that led to the varied outcomes is still unclear.

**Figure 1:**
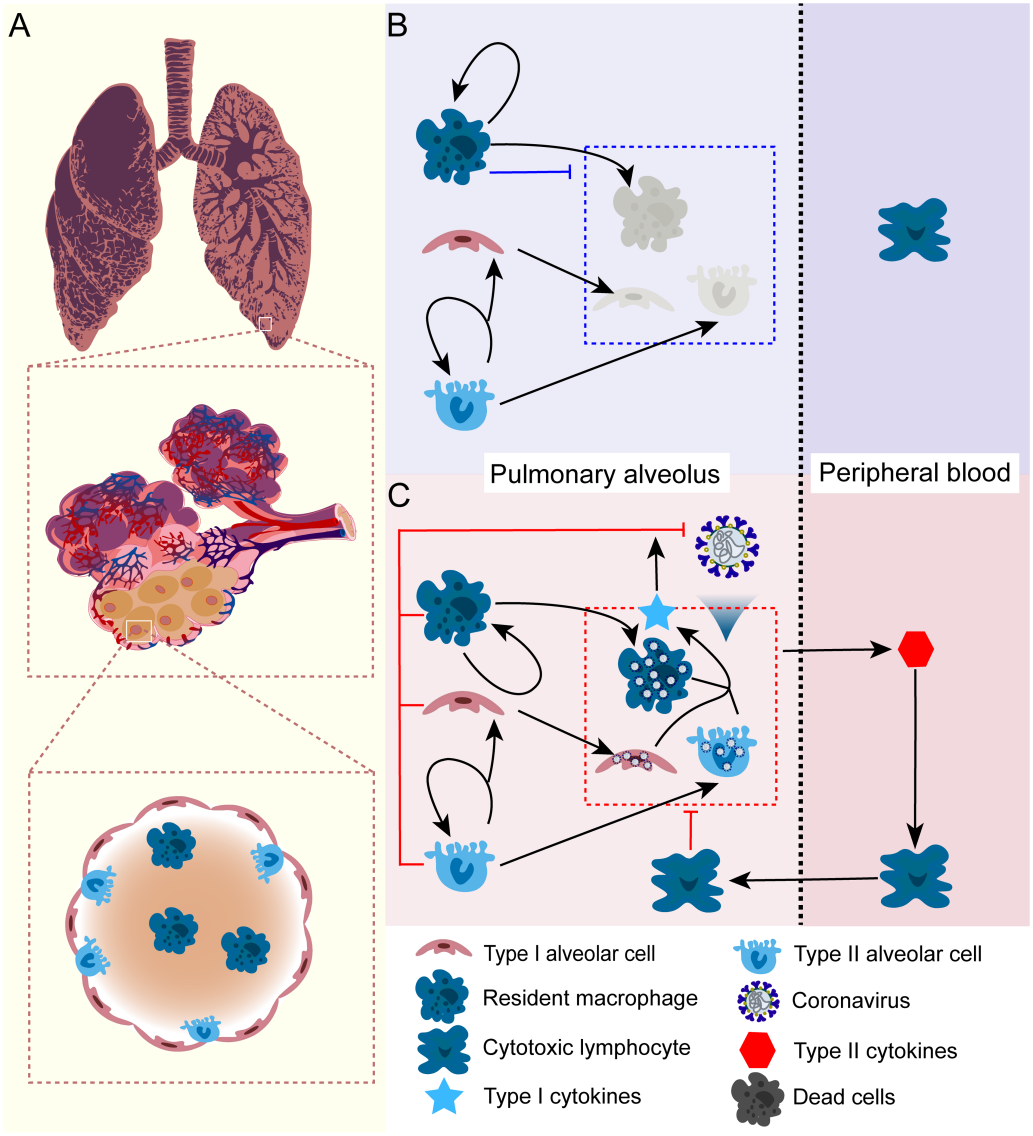
The schematic illustration of cellular dynamics of pulmonary alveolus in health condition and under SARS-CoV-2 infection. **(A)** The anatomic structure of the pulmonary alveolus. The alveoli consist of two types of cells: type I ( $C_{I}$ ) and type II ( $C_{\mathrm{II}}$ ) cells. Type I cells maintain the alveolar structure and gas-exchaging function, type II cells are mainly responsible for cell renewal of both types. In addition, there is a tissue-specific macrophage ( $M_{\phi }$ )that reside inside the alveolus. **(B)** In health lung, type II alveolar cells proliferate itself and differentiate into type I cells in order to maintain the structure of the lung. The resident macrophage maintains constant division inside alveolus. The resident macrophage can also clear the apoptotic alveolar cells and inhaled microbes, which substantially inhibits local inflammation inside the lung. **(C)** When exposed to COVID-19, alveolar cells including resident macrophage will be infected. Viral infection can substantially impairs the cell division and results enhanced pyroptosis and cell death. Unstoppable infection and cell death release large quantity of cytokines and chemokines into peripheral blood, which elicits proliferation of lymphocytes and attracts them to infection site and sometimes cause lung destruction and sever symptom. It is important to note that this only represents a conceptual schematic for our mathematical framework, but it does not show new experimental data. It demonstrates the biological presumptions that guide our model, including immune response activation during infection, alveolar cell turnover, and macrophage function.

In order to gain a quantitative understanding of COVID-19 progression, we established a simple theoretical framework to investigate how various factors collectively contribute to the heterogeneity of disease progression and treatment outcomes. In doing so, we first analyzed the cellular dynamics of pulmonary cells in the lung (see Figure 1), then built a mathematical framework to capture the cellular dynamics of health and virus-infected alveoli. Pulmonary alveoli are consists of two types of cells, type I and type II cells. Pulmonary renewal is sustained by the division of type II cells. Resident macrophages scatter on the inner surface of the alveoli and remove invading pathogens, dead cells, and inhibit inflammation (Divangahi et al., 2015). Such dynamics maintain pulmonary homeostasis. When infected by COVID-19, the alveolar cells suffer impaired growth and division. We assume that viral infection will not immediately cause cellular death and apoptosis. Instead, it will deplete heath cells with a measurable rate and render the infected cell into apoptotic cells, whose cytokines releasing will cause severe immune-mediated pathology with the presence of cross-reactive cytotoxic T cells. With the mechanistic framework, we then modeled treatment with viral entry inhibitors, viral proliferation inhibitors, cytokines inhibitors, and combined treatment. We analyzed how dosage and treatment windows would affect the outcome and recovery variation. With this framework, we hope to establish a dynamic process to explain how the virus and pulmonary cells interact and determine the outcome of an infection. Moreover, this model can provide theoretical guidance for the treatment of SARS-CoV-2 and other virus-associated disease.

## Methods

### Cellular dynamics of alveolar cells in healthy lung

We first look into how alveolar homeostasis is maintained in healthy conditions in order to comprehend the dynamics of pulmonary pathogenesis under COVID-19. There are mainly two types of alveolar cells that compose the alveoli: type I alveolar cells and type II alveolar cells (Nalayanda et al., 2009). Type II cells proliferate and differentiate into type I cells in a certain proportion of ∈. Thus, the dynamics of type II and Type I cells, $C_{\mathrm{II}}$ , $C_{I}$ , can be written as:


(1) $$\frac{dC_{\mathrm{II}}}{\mathrm{dt}}=\alpha (1-\in )C_{\mathrm{II}}-\delta_{x}C_{\mathrm{II}}$$

(2) $$\frac{{\mathrm{dC}}_{I}}{\mathrm{dt}}=\alpha \in C_{\mathrm{II}}-\delta_{x}C_{I}$$

We assume that all types of cells have the same rate of aging or apoptosis, $\delta_{x}$ . The resident macrophages on the surface of alveoli are able to self-renew with rate β . We have the dynamics of resident macrophages, $M_{\phi }$ :


(3) $$\frac{{\mathrm{dM}}_{\phi }}{\mathrm{dt}}=\beta (1-M_{\phi }/K)M_{\phi }-\delta_{x}M_{\phi }$$

where K represents the carrying capacity of macrophages in the alveolar environment. These macrophages play important roles in removing invading pathogens and apoptotic cells, as well as inhibiting inflammation (Byrne et al., 2015) (Figure 1B). Aged or apoptotic cells can be removed by macrophages at a certain rate $\phi_{C}M_{\phi }$ . We have the dynamics of apoptotic cells:


(4) $$\frac{{\mathrm{dC}}_{x}}{\mathrm{dt}}=\delta_{x}(C_{\mathrm{II}}+C_{I}+M_{\phi })-\phi_{C}M_{\phi }C_{x}$$

Notably, if ${\mathrm{dC}}_{x}/\mathrm{dt}<0$ , The apoptotic cells can always be removed, and the homeostasis of alveolar cell growth is maintained. In summary, standard cell population dynamics serve as the foundation for the equations that describe alveolar cell homeostasis in a healthy lung. Alveolar epithelial cell renewal is modeled by Equations 1, 2, in which type II cells self-proliferate and develop into type I cells at a specific ratio. Equation 3 captures the self-renewal of resident alveolar macrophages, while Equation 4 depicts the removal of apoptotic cells through macrophage-mediated phagocytosis. Previous observations in biology support these mechanisms. We simulated the system under healthy, non-infected settings (i.e., no viral load) to confirm the model’s accuracy. We saw that the cell populations stabilized and achieved a steady state, which reflected normal alveolar homeostasis.

### The dynamics of viral infection and disease progression

Due to the wide distribution of angiotensin-converting enzyme 2 (ACE2) in tissues and organs, SARS-COV-2 can enter and infect most of, if not all, the tissues and organs (Vabret et al., 2020). Alveolar cells are among the first to encounter the virus during the course of infections. We divided alveolar cells into three groups when infected with the COVID-19: healthy cells, infected cells, and dysfunctional cells. With the above dynamical model of healthy alveoli, we assume that only non-infected type II cells can divide. Infected cells are still able to maintain the pulmonary structure and function of gas exchange despite losing their ability to proliferate. If the viral load decrease, these infected cells are able to revert to healthy cells. We define dysfunctional cells as a group of cells that are unable to exchange gases. These cells accumulate as a result of virus-induced pyroptosis, immunopathogenesis, and subsequent fluid infiltration. In particular, the infected alveolar cells can secrete interferon to neighbouring cells and enhance their antiviral capacity, which can substantially reduce viral load. In case of uncontrolled viral infection, cytokines are released from dead and dysfunctional cells into the peripheral blood, attracting cytotoxic lymphocytes to the infection site. The resulting massive lung damage substantially impairs gas exchange in the lung and causes severe clinical manifestations (see Figure 1C). Specifically, we decompose the entire process of infection and manifestation as follows:

#### Viral infection

All alveolar cells are infected by the virus with same rate σV , at which uninfected cells will be removed. In addition, infected cells can recover depending on the viral load. We have the dynamics of uninfected cells, $C_{\mathrm{II}}$ , $C_{I}$ and $M_{\phi }$ :


(5) $$\frac{dC_{\mathrm{II}}}{\mathrm{dt}}=\alpha (1-\in )C_{\mathrm{II}}-\mathrm{\sigma V}C_{\mathrm{II}}+\lambda_{1}r(1-f(V))C_{\iota }$$

(6) $$\frac{{\mathrm{dC}}_{I}}{\mathrm{dt}}=\alpha \in C_{\mathrm{II}}-{\mathrm{\sigma VC}}_{I}+\lambda_{2}r(1-f(V))C_{\iota }$$

(7) $$\frac{{\mathrm{dM}}_{\phi }}{\mathrm{dt}}=\beta (1-M_{\phi }/K)M_{\phi }-{\mathrm{\sigma VM}}_{\phi }+\lambda_{3}r(1-f(V))C_{\iota }$$

The dynamics of alveolar cells during viral infection are described by Equations 5–7. While the recovery rate term enables infected cells to return to a healthy condition when the viral load drops, the infection rate term (σV) simulates viral entry into healthy alveolar cells. General virological concepts like ACE2-mediated entrance and immune-regulated viral suppression serve as the foundation for these calculations. This abstraction makes it possible to analyze common characteristics among coronaviruses, even though the model does not take into consideration variations among viral strains. We modeled a range of infection outcomes, such as viral clearance, mild progression, and severe disease, in order to validate these equations. These model results supported the biological relevance of the model by matching clinical trends seen during the COVID-19 pandemic.

Here, we assume that infected cells are not able to proliferate. The apoptotic rate of healthy cells in an uninfected condition, denoted as $\delta_{x}$ , is comparably low, and we assume that $\delta_{x}\ll \mathrm{\sigma V}$ , thus can be neglected. If the virus inside the cell can be suppressed and eliminated, the infected cells will turn into healthy ones at a rate r(1−f(V)) , where r is a rate parameter, and $f(V)=\frac{V^{n}}{V^{n}+\Theta_{V}^{n}}$ . V is the viral load and $\Theta_{V}$ is the half-saturated threshold of viral load, n is the Hill coefficient. $\lambda_{i}$ is the ratio factor of each type of cell, with $\sum \lambda_{i}=1$ .

#### Cell death and removal

SARS-CoV-2 infection does not immediately lead to cell death, but can lose ability of the division to divide, at a rate denoted as $\delta_{x,\mathrm{vir}}$ , due to viral pathogenesis. In the case of cytokines storm, these infected cells, $C_{\iota }$ , can be directly killed by cytotoxic lymphocytes $C_{T}$ , if present, due to the presence of viral antigens on the surface. The removal rate denoted as $\phi_{T}C_{T}$ . The dynamics of infected cells are described in Equation 8 as:


(8) $$\begin{array}{l} \frac{{\mathrm{dC}}_{\iota }}{\mathrm{dt}}=\mathrm{\sigma V}(C_{\mathrm{II}}+M_{\phi }+C_{I})-\delta_{x,\mathrm{vir}}C_{\iota }-\phi_{T}C_{T}C_{\iota }(\frac{I_{l}^{n}}{I_{l}^{n}+I_{l,50}^{n}})\\ -r(1-f(V))C_{\iota } \end{array}$$

Dead cells, $C_{x}$ , are accumulated from the killed infected cells and can be removed by uninfected macrophages. The dynamics are described in Equation 9 as:


(9) $$\frac{{\mathrm{dC}}_{x}}{\mathrm{dt}}=\phi_{T}C_{T}C_{\iota }(\frac{I_{l}^{n}}{I_{l}^{n}+I_{l,50}^{n}})+\delta_{x,\mathrm{vir}}C_{\iota }-\phi_{C}M_{\phi }C_{x}(\frac{I_{f}^{n}}{I_{f}^{n}+I_{f,50,C}^{n}})$$

#### Viral replication and immune response

The virus replicates inside the infected cells. We calculate the virus growth rate as the average number of viral particles, so the rate of virion production, as shown in Equation 10, is given by ${\mathrm{\gamma C}}_{\iota }$ , where population size ( $C_{\iota }$ ) is the number of infected cells, γ is the number of viral particles each cell produces. Viral replication can be diminished by cellular regulation through interferon signals (Major et al., 2020). The viral growth can be written as:


(10) $$\frac{\mathrm{dV}}{\mathrm{dt}}={\mathrm{\gamma C}}_{\iota }-\phi_{V}(\frac{I_{f}^{n}}{I_{f}^{n}+I_{f,50,V}^{n}})(C_{\mathrm{II}}+C_{I}+M_{\phi })V$$

Where $\phi_{V}$ is the rate at which healthy cells clear virus. $I_{f}$ is the type I cytokines, such as interferon shown in Equation 11, which is produced by infected cells, and removed at a constant rate $\delta_{I}$ :


(11) $$\frac{{\mathrm{dI}}_{f}}{\mathrm{dt}}=\rho_{f}(\frac{C_{\iota }^{n}}{C_{\iota }^{n}+\Theta_{\iota }^{n}})-\delta_{I}I_{f}$$

Massive infection and cellular dysfunction cause a cytokine storm and a self-targeting immune response. These cytotoxic immune cells mainly originate from peripheral blood and are attracted to the infected site by cytokines. Here, we do not consider the loss of function of cytotoxic immune cells when infected by the virus, and only consider functional cytotoxic immune cells, $C_{T}$ , that arrive at the infected site. The rate equation for type II cytokines can be written as Equation 12:


(12) $$\frac{{\mathrm{dI}}_{l}}{\mathrm{dt}}=\rho_{l}(\frac{C_{x}^{n}}{C_{x}^{n}+\Theta_{x}^{n}})-\delta_{I}I_{l}$$

$\Theta_{i}$ ( i = { ι , x }) is the half-saturated threshold of dead and dysfunctional cells, and n is the Hill coefficient.

### Model analysis and implementation of *in silico* treatment

In order to specifically study the impact of kinetic factors on the pathogenesis and treatment of COVID-19, we performed detailed numerical simulation and analysis of the model. We define the “*in silico* clinical manifestation” based on the temporal patterns of cell numbers and viral load. During the course of infection, when the number of infected cells is less than the number of healthy cells, we define the time interval as the asymptomatic phase; when the number of infected cells is exceeds the number of healthy cells, we define the time interval as the symptomatic phase. When the number of dead and dysfunctional cells is more than the functional cells, we define the time interval as severe phase. In order to study how the factors collectively determine the infection outcomes, we tested the effects of several parameters, including alveolar cell renewal, macrophage phagocytosis, and interferon production, on disease progression.

Various strategies have shown potency in treating COVID-19, including preventing viral entry (Hoffmann et al., 2020), inhibiting viral replication with antiviral drugs (Hung et al., 2020), directly neutralizing viral particles through monoclone antibodies (Zhu et al., 2020; Jiang et al., 2020) or convalescent plasma (Simonovich et al., 2021; Subbarao et al., 2020), and eliminating inflammatory cytokines in peripheral blood (Zhu et al., 2020; van der Wijst et al., 2021). In the above dynamical system, we modeled and parameterized several critical steps of disease progression. The three most important steps are viral entry, with rate σ , viral replication, with rate γ and cytokines production, with rate ρ . We further conducted *in-silico* investigation to understand how the clinical treatments could mitigate disease progression and promote recovery. We simulated three main treatment strategies: reducing viral load, inhibiting viral entry and inhibiting over-reactive immune response induced by cytokines. For simplicity and proof-of-principle, we do not consider the pharmacodynamics and pharmacokinetics, instead assuming constant concentration and manipulating the rate parameters. For example, changing the parameter γ into 0.1 γ means the rate of viral replication is reduce to 10% of its maximal rate. To test the timing of treatment implementation, we changed the parameter values at different time point in the course of disease progression. To test the effect of these treatment strategies in a cohort of patients with heterogeneous disease progression, we first tuned the three parameters above and generated an “in silico disease manifestation” in individual patient according to the above definition. In simulating cohort treatment, we created a 70% severity rate in 2000 patients by randomly setting the rate of phagocytosis $\phi_{C}$ , infection rate σ and rate of cytokine production ρ with distributions (10^−6^ and 10^−2^), (10^−9^ and 10^−7^) and (10^−5^ and 10^−3^), respectively. We calculated the average time of the asymptomatic phase, symptomatic phase, and severe phase for the 2000 patients. We then introduced the treatment strategies with different regimes and their combination at different phases and accessed the recovery rate and cure time. Recovered cases were collected when viral load is less than 10^3^ at the end of treatment. All calculations and simulations were performed in R 4.0.4.

## Results

### A mechanistic model of SARS-CoV-2 infection and disease progression

In this study, we built a mechanistic model that mainly captures the dynamics of alveolar cells, viral infection, and cellular immune responses mediated by interferons, cytokines, and cytotoxic T cells (Figure 1). Our model focuses on the dynamics of these interactions and provides insights into the immune response against SARS-CoV-2 infection in the lungs. It should be noted that our model is based on the assumption that there is a physical isolation between the pulmonary alveoli and peripheral blood. This physical isolation can be weakened when viral infection cause cellular pyroptosis and releases cytokines. This indicate that pulmonary alveoli function independently in terms of immune response against SARS-CoV-2 infection, as they have resident macrophages that eliminate invading pathogens. Additionally, viral infection can be also eliminated by cellular immunity mediated by interferons produced by neighbouring cells (Figure 1C). When pulmonary infection cannot be controlled by this mechanism, the pyroptotic cells caused by the infection will release other specific cytokines to attract cytotoxic lymphocytes to the infection site, which might cause severe symptoms.

### The dynamics of alveolar cells, viral load and progression of COVID-19

**Figure 2:**
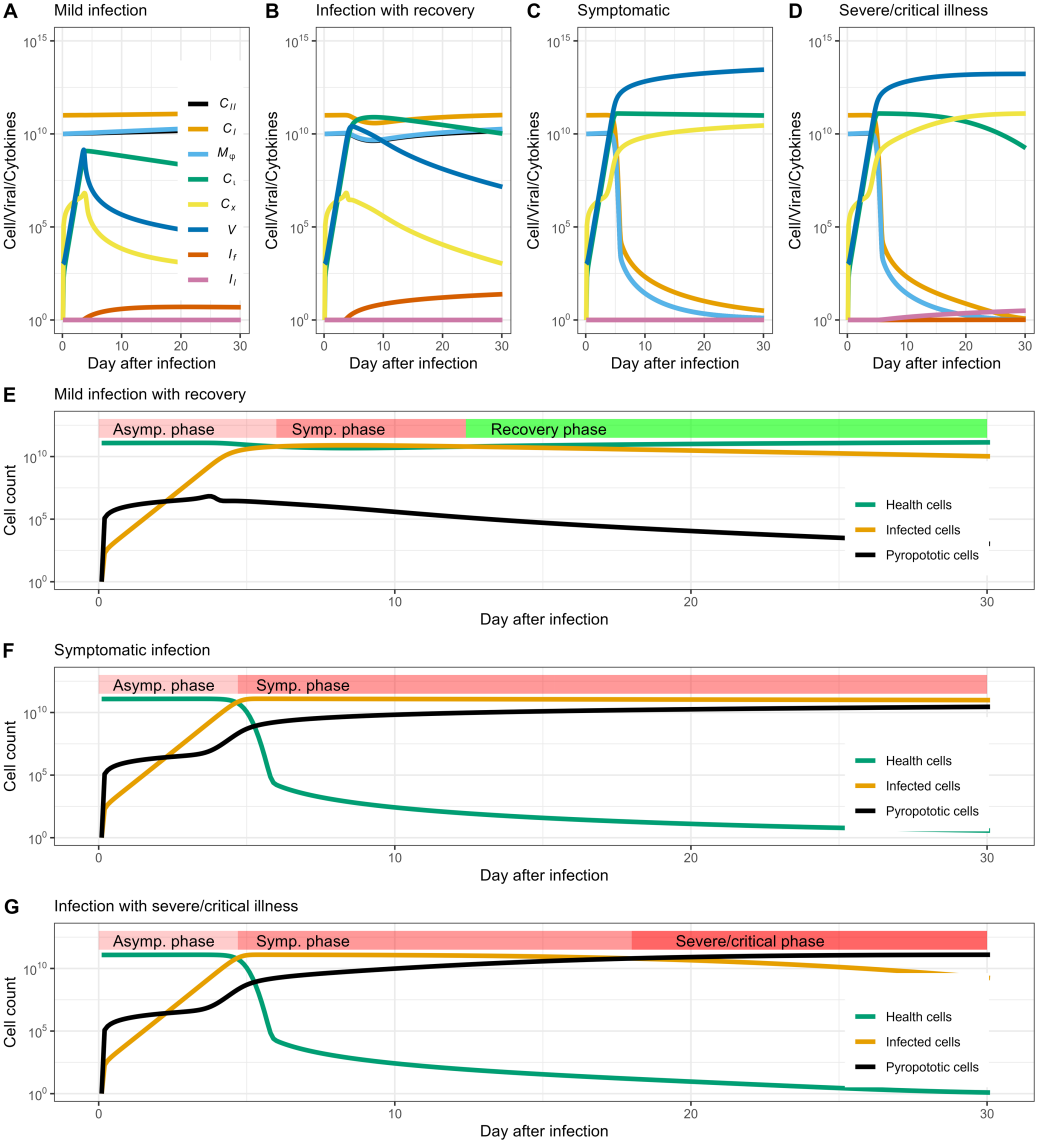
Cell and virus dynamics capture disease progression. **(A–D)** Show the dynamic of cells and virus in the patient that mark the heterogeneity of infection outcomes: no infection, infection with mild symptoms and infection with severe symptoms, respectively. When the patient enters the mild stage, the number of cells with normal functions in the body decrease significantly, and the infected cells and viral load increase; in the severe stage, the infected cells and viral load exceed normal cells, and a large number of cells die or loosing function of gas exchanging. **(E,F)** Show the changes in healthy cells, infected cells, and cell populations that have lost function in different periods. When the number of infected cells can be gradually reduced, and the number of pyroptotic cells remains stable and does not rise, the patient enters the recovery period instead of developing into a severe illness.

Here, we first analyzed the cellular dynamics of alveolar cells in both healthy and infected condition (Figure 1), then connected the temporal variation of alveolar cells with disease progression and symptom manifestation. Viral infection does not immediately manifest symptoms if the inflammatory response is not initiated. In the case of COVID-19, severe symptoms are mostly the consequences of over-reactive immunity that targets the patient’s tissues and organs. A stronger cellular response mediated by interferon can more quickly eradicate the virus inside the cells. Lymphocyte’s profiling showed that cross-reactive T cells are mainly responsible for the severe symptoms. In the simulation, we thus tuned two parameters, $C_{\text{TO}}$ and ρ, which represent initial cross-reactive cytotoxic T cells and rate of interferon production. The dynamics revealed in our model defined three different statuses corresponding to real-world infection: (1) no infection, (2) infection with mild symptoms, and (3) infection with severe/critical illness (Figures 2A–C), respectively.

In Figure 2A, we define this scenario as “no infection” because the viral population is rapidly eliminated and almost no cells are infected or killed. This may be due to fast cellular immune response ( ρ = 10^−1^) and low availability of cross-reactive cytotoxic lymphocytes ( $C_{\text{TO}}$ = 1). When the infection demonstrates no or mild symptoms ( $C_{\text{TO}}$ = 1, ρ = 10^−4.8^), the virus propagates and infects some alveolar cells, but does not kill these cells or cause an overwhelming inflammatory response, showing the typical characteristics of a self-limiting disease (Figure 2B). Specifically, we consider the recovery process as an intrinsic process, where tissue damage is associated with viral load. As the viral load decrease, the lung cells automatically recover. In clinical cases, the loss of function in lung cells is mainly caused by fluid infiltration, and the lung cell function can be retained once the infiltrated fluid is absorbed. When infection develops severe symptoms ( $C_{\text{TO}}$ = 10^, ρ = 10^−5^), viral proliferation and subsequent pathogenesis cause cell death and elicit a strong immune response. Alveolar cells are infected by viral particles and lose function due to excessive inflammatory response. In clinical manifestations, this case can be characterize by massive fluid infiltration in the lungs. Based on the cellular and viral dynamics, our model further captures the stages of clinical manifestations in individual patients, such as the asymptomatic phase, symptomatic phase and recovery phase/severe phase (Figures 2D,E). In particular, our model predicts that the transition from symptomatic phase to severe phase is quite swift (Figure 2E), usually lasting 1–2 days in early cases infected by earlier viral strains (Guan et al., 2020). Using parameter values from previously published research, such as estimations of viral clearance rates, cytokine production, and alveolar cell dynamics (as summarized in Table 1), Figure 2 depicts simulated trajectories of COVID-19 progression. In order to replicate the normal clinical course of COVID-19, which typically consists of the incubation phase (days 0–7, characterized by increase of infected cells), the symptomatic phase (days 7–14, pyroptotic cell are accumulating), and the possible transition to severe or critical disease (days 14–30, pyroptotic cell outnumber healthy cells), we selected a 30-day simulation period. Further simulations that lasted longer than 30 days revealed no qualitative variations in the course of the disease, confirming that this period is a suitable and effective window to depict the entire infection cycle.

**Table 1:** Key parameter values used in the simulations.

Parameter/states	Description	Value/value space	Sources
$C_{\mathrm{II}}$	Type II alveolar cells	10^10^ (cells)	Nalayanda et al. (2009) and Byrne et al. (2015)
α	Renewal rate of Type II alveolar cells	10^−3^ (cell^−1^.day^−1^)	Nalayanda et al. (2009)
$C_{I}$	Type I alveolar cells	10^11^ (cells)	Nalayanda et al. (2009) and Byrne et al. (2015)
∈	Ratio of Type I cells generated by proliferation of Type II cells	0.8 (−)	
$M_{\phi }$	Resident macrophages	10^9^ (cells)	Byrne et al. (2015) and Wallace et al. (1992)
β	Renewal rate of resident macrophages	10^−3^ (cell^−1^.day^−1^)	Byrne et al. (2015)
$\delta_{x}$	Rate of health cells turned into dead cells in uninfected condition	10^−15^ (cell^−1^.day^−1^)	
$C_{x}$	Dead cells	Set to 0 as initial value	
$\phi_{C}$	Removal rate of resident macrophages	10^−3^ (cell^−1^.day^−1^)	Li et al. (2022)
V	Viral load	Varied initial values	
γ	Viral proliferation in infected cells	10^−3^ (V.cell^−1^.day^−1^)	Zhou et al. (2023)
$C_{\iota }$	Infected cells	Set to 0 as initial value	
σ	Infection rate of virus	10^−8^ (cell^−1^.day^−1^)	Shapira et al. (2022)
$\lambda_{i}$	Ratio factor of each type of cell	$\lambda_{1}=\lambda_{3}=0.1$ ; $\lambda_{2}=0.8$ (−)	
r	Rate of infected cells turned into healthy cells when virus is cleaned	0.1 (−)	
$\delta_{x,\mathrm{vir}}$	Death rate of infected alveolar cells	10^−4^ (cell^−1^.day^−1^)	
$\phi_{T}$	Killing rate of cytotoxic lymphocytes	10^−4^ (cell^−1^.day^−1^)	
$C_{T}$	Cytotoxic lymphocytes	Varied initial values	Schulien et al. (2021)
I	Cytokines	Varied initial values	Broggi et al. (2020)
$\phi_{V}$	Rate that health cells clear virus	10^−5^ (cell^−1^.day^−1^)	
$\delta_{A}$	Removal rate of interferon	10^−3^ (day^−1^)	
ρ	Rate of interferon production	10^−4^–10^−1^ (−)	
$C_{T0}$	Cytotoxic immune cells	10^1^–10^4^ (cells)	
ρ	Rate of cytotoxic lymphocytes moving to infected site	–	
*K*	Capacity for resident macrophages	10^10^	Wallace et al. (1992)
*n*	Hill coefficient	2	

### Decomposing the complex factors contributing to the infection outcomes

With the above definition of infection outcomes, we further investigated how the host, pathogen and other factors collectively determine the outcome of the infection. We identified and analyzed the parameters of four main factors that related to the infection process: the rate of viral infection, the rate of viral clearance, the rate of pulmonary cell proliferation, the rate of phagocytosis of resident macrophages, and the availability of cross-reactive cytotoxic T cells.

**Figure 3:**
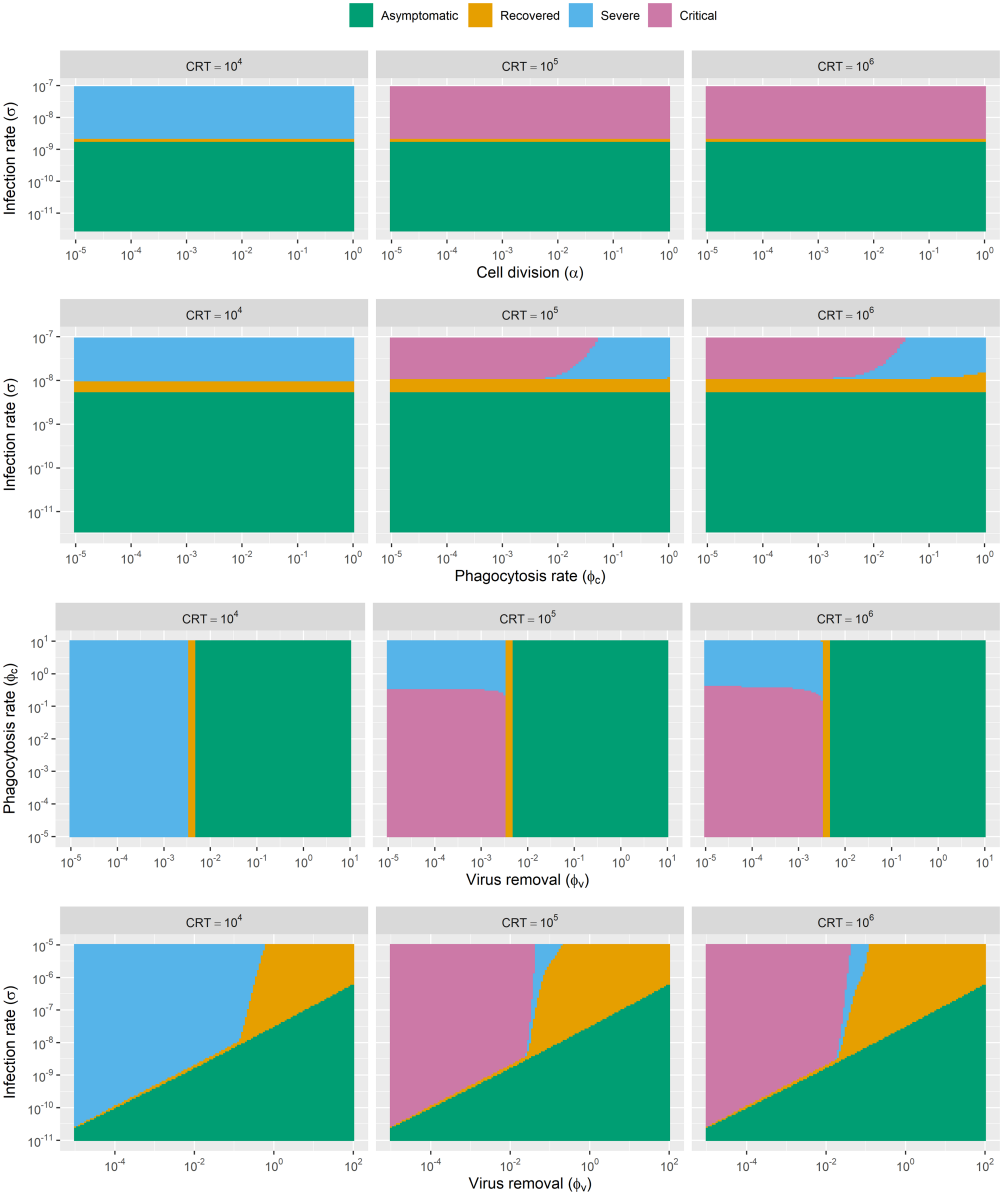
The phase diagram of infection outcomes with range of parameters of rate of viral infection, rate of viral clearance, rate of pulmonary cell proliferation and rate of phagocytosis of resident macrophages. Simulations are based on SARS-CoV-2 parameters, though the model framework may apply to other coronaviruses with similar infection dynamics.

By manipulating two of these parameters at a time, we generated phase diagrams (Figure 3) for each pair of parameters. In this way, we were able to know how a single parameter as well as parameters pairs decide the trajectory of disease progression. Consequently, we generally find three to four different infection outcomes across different parameters spaces (Figure 3). The simulation results showed that the rate of pulmonary cell proliferation does not change the consequence of infection within a large rang of parameter space from 10^−5^ to 1. Additionally, our modelling results indicated that phagocytosis has little impact on disease progression in certain parameter spaces, as the parameters space (from 10^−5^ to 10^−2^) explored in our simulation did affect the infection outcomes. When the phagocytosis rate exceeds 10^−2^, it results better outcome of infection. It is intuitive that higher infection rate leads to worse disease outcomes. When the infection rate increases from 10^−9^ to 10^−8^, the *in-silico* “patients” exhibit more severe symptoms. Similarly, an increase in virus removal in early stage of infection drastically increase the chance of recovery without medical interference/interventions. As the rate of clearance increases, the infection outcomes change from “infection with recovery” to “asymptomatic infection.”

Besides, we also found that the disease development trajectory of the “patients” was closely related to the availability of cross-reactive lymphocytes. In our model, this is the key parameter that determines whether the disease will develop into severe and critical stages. We explored three parameters for cross-reactive lymphocytes (10^4^, 10^5^, 10^6^). Infected “patients” with high initial level of cross-reactive lymphocytes are more likely to develop severe symptoms and fail to recover from the infection.

### Medical intervention: strategy and time window

A variety of treatment strategies have been applied to fight against the virus and its associated pneumonia. Many of these strategies provided plausible solutions for clinical cases. We conducted a cohort of 2000 random simulations with different disease development trajectories by tuning the parameters of viral infection rates, cell proliferation rates, and cross-sensitive T cells. We set the average ratio of patients with critical symptoms at about 30% in the cohort received no medical intervention. The average recovery time was approximately 80 days, with the standard that no virus being detected in patients. When adopting different treatment plans to intervene and treat patients, we used the simulation results as a reference to evaluate the effects of the treatment plans and timing of intervention accordingly.

**Figure 4:**
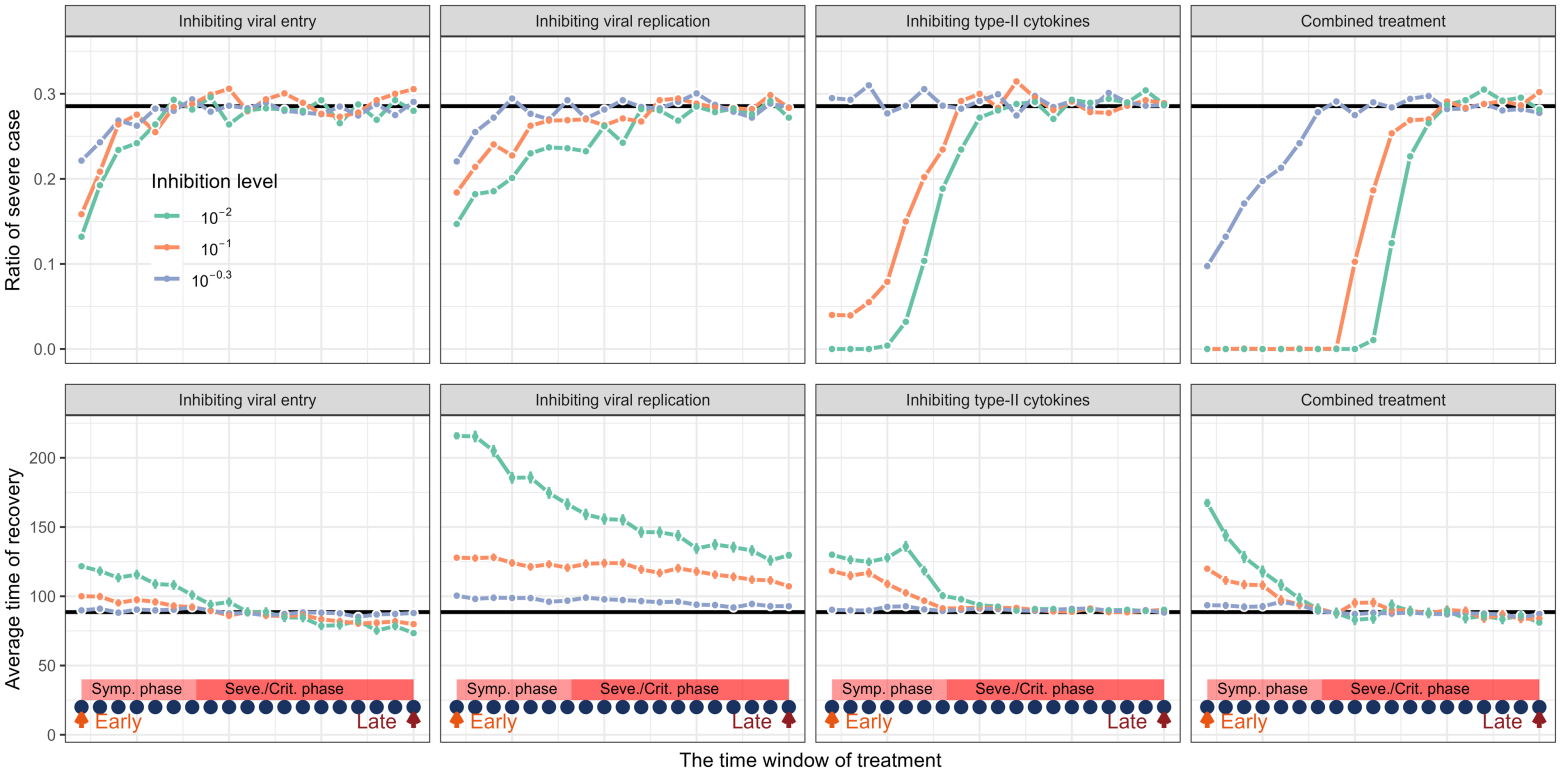
The impact of different medical interventions along the time of disease progression. The upper panel represents the proportion of severe cases and the lower panel represents the rate of recovery before and after treatment. The horizontal black lines are their average values of a cohort of in-silico “patient” before treatment. The dark blue dots represent the time points when the intervention is implemented. The treatment window spans from symptomatic phase to severe or critical phase. Simulation of treatment effects initiated at three time points: early (day 5, onset of symptomatic phase), intermediate (day 15, disease progression phase), and late (day 25, severe phase). Treatments continued thereafter. Results show that early treatment significantly improves outcomes.

In Figure 4, we found remarkable differences in recovery rate and in-hospital duration when implementing these different strategies. In general, the simulation results indicated that medical intervention should begin as early as possible in the course of infection. We found that earlier intervention results in higher treatment success but longer in-hospital duration. When applying a single treatment strategy, the medical intervention started at the onset of infection or “Symptomatic phase,” by our definition can nearly “cure” half of the infected patients. Moreover, it is worth to point out that applying single treatment strategy is unlikely to cure all the infected patients. Our simulations support the combined treatment as the best strategy in treating COVID-19. Combined treatment with the three individual methods described above resulted in a considerably higher chance of treatment success and shorter in-hospital duration. Additionally, our simulation showed that implementing of treatment in the late stage of infection still leads to worse outcomes. There is also a “trade-off” when implementing these treatment strategies, as early treatment results in longer in-hospital duration (Figure 4).

## Discussion

SARS-CoV-2 infection typically begins in the lungs before disseminating to the peripheral blood and various organs and tissues. The viral load in the lungs is influenced by the infection rate, turnover rate of alveolar cells, and cellular immune response. Our study presents a mechanistic model that incorporates these dynamics to comprehensively understand the progression of COVID-19 and its treatment outcomes. Our model highlights several key separate and coherent steps that determine the trajectories and outcomes of the infection. First, our model assumption is based on the physical barrier between pulmonary environment and peripheral blood, which ensures that the lungs can maintain a self-sustained immune mechanism to eradicate the infection. Due to the fact that the physical barrier can separate the pulmonary immunity and humoral immunity, the signal of infection can be barely transmitted to the peripheral blood at the early stage of infection. When healthy lungs are exposed to COVID-19, alveolar cells, including resident macrophages become infected. Viral infection can severely impair cell division, cause loss of cell function, and leads to cell death due to pulmonary embolism if the infection is not controlled. Then, unstoppable viral infection and cell death cause a large number of cytokines and chemokines to be produced and released into the peripheral blood, thereby attracting cytotoxic lymphocytes to the infected site. This may result in severe symptoms or multiple organ dysfunction with similar mechanism.

By integrating above important assumptions, our model captures many features of disease progression and clinical manifestations. One of the typical ones is the sharp transition from mild to severe or critical symptoms, which is not observed in conventional pneumonia caused by other viruses. This sharp transition was commonly observed in case at the beginning of pandemics in Wuhan (Guan et al., 2020). Our model indicates that this abrupt transition is actually caused by the massive release of cytokines from infected cells and the influx of immune factors in the peripheral blood after the collapse of physical barrier between organs and the blood, with the presence of cross-reactive cytotoxic T cells (Mateus et al., 2020; Kaplonek et al., 2021). Similar modelling assumptions and results have also been implemented and obtained, showing that the IFN response is essential to modulate the transition from asymptomatic to symptomatic stages in different patients (Wang et al., 2021). For proof of principle, however, our model does not explicitly cover dynamics of all components of immune cells and details of cytokines (Zhou et al., 2023). Also, we did not consider the other factors, such as collateral bacterial infection, that contributes to the sharp transition in disease progression (Li et al., 2022).

Despite the spatial differences in different organs controlling the heterogeneity of disease progression, we tuned different parameters and identified several key factors that contribute to the variable disease trajectory. The simulation results have shown that lower infection rates, higher phagocytosis rates and cytokine production all lead to less severe symptoms without treatment. Different combinations of parameters resulted in the phase plain of the symptoms, which is the origin of heterogeneity of disease patterns in individual patients. Infection rate is defined as how likely a virion can enter and infect a cell, which is determined by the receptor angiotensin-converting enzyme 2 (ACE2) for SARS-COV-2. For example, clinical records showed that people in younger age are less likely to develop severe symptoms when infected (Guan et al., 2020; Parri et al., 2020). This may be partially due to the fact that pulmonary epithelia in younger people express less viral receptors (Bickler et al., 2021; Peng et al., 2021; Silva et al., 2023). Blocking these receptors can achieve much lower infection rate (Hoffmann et al., 2020; Cheng et al., 2020; Shapira et al., 2022). In addition, younger people may have higher turn-over rate of the pulmonary cells. Other factors, such as smoking life style, also affect the expression of viral receptors and thus cause different infection outcomes I smokers and non-smokers as smoking increase the expression of ACE2 (Smith et al., 2020). Besides, our model also captured the dynamics showing that an increase in the rate of phagocytosis in lower respiratory tract can result in better infection outcome. As the resident macrophages can substantially reduce neutrophil-driven inflammatory damage (Uderhardt et al., 2019), also inactivate CD4 T cell mediated inflammatory immune response (Divangahi et al., 2015; Blumenthal et al., 2001; Schneider et al., 2014). In addition, cytokine is one of the most important factors that inhibits the intracellular infection of virus. For example, IFN deficiency could increase host susceptibility to various pathogen infection (Divangahi et al., 2015). But the cytokines may collectively influence the antiviral capacity in a very complex way (Sposito et al., 2021). It is notable that cross-reactive cytotoxic lymphocytes are one of the critical factors that cause severe symptoms in coronavirus infection (Ramaswamy et al., 2021; Schulien et al., 2021). These lymphocytes are the first wave that was attracted by the cytokine storm in the lungs. Our model showed that pre-existence of cytotoxic lymphocytes (such as CD8^+^) can significantly increase the chance developing severe symptoms in patients.

Various strategies with different classes of drugs have been developed to treat SARS-COV-2 infection at its different developmental stages. This includes drugs that inhibit viral entry (Shapira et al., 2022) and replication (Cao et al., 2020; Hu et al., 2021; Mahévas et al., 2020). Our model and results of *in-silico* treatment showed that different treatments can result in variable outcomes. Notably, earlier treatment could results in better outcomes based on the simulation. During the pandemic, many drugs have been tested in clinical trials, such as lopinavir-ritonavir, a compound used to competitively inhibit viral RNA polymerase and block viral replication and transmission. Clinical trials showed that drugs applied only in the early stage of infection can result to better treatment outcomes (Cao et al., 2020; Hung et al., 2020). This also happen to the drug hydroxychloroquine, that inhibits ACE2 to prevent the Covid-19 from entering the cell (Geleris et al., 2020; Funnell et al., 2020). Although, inhibition of inflammatory cytokines, such as IL-6, can significantly reduce the probability of developing severe symptoms, treatment with Tocilizumab in later stage of the severe cases still cannot reverse the disease progression (Stone et al., 2020). Also, drug combination is widely applied in treating covid-19. Our modelling results showed that combined drug regimes will significantly increase the treatment success when applied at the early stage of disease progression. Similar results have been obtained by theoretical studies using different modelling techniques (Dodds et al., 2021).

Choosing the right time window and drug combinations for treating COVID-19 and other similar disease can significantly improve treatment effectiveness and reduce total medical costs. However, potential challenges still exist when such an optimal strategy is deployed. Our model has suggested the cause of the heterogeneity of disease progression and treatment outcomes, in which the optimal strategy can be explored and implemented. The spatial heterogeneity that caused by the physical isolation between lung and peripheral blood suggests that the disease progression is actually a one-way process, which can be hardly reversed especially when disease develops to late stages. Also, many clinical investigations also have demonstrated that the only way to cure the COVID-19 is to prevent it before it’s getting worse. In addition, this optimal management also rely on proper detection of disease signals. This is particularly important when there is a need for reducing the overall treatment cost in case of an explosive outbreak. Another challenge exists in the current treatment protocols. In many cases, treatment only implemented when the symptom manifests. Our model results are also in line with some prophylactic treatment strategies should be consider for reducing the total costs as the start time of treatment matters the overall outcomes (van de Veerdonk et al., 2022; Plaçais et al., 2022).

There are still limitations in our mathematical framework. Despite our model having captured some important features of disease progression and also give practical suggestions for treatment, detailed clinical data and physiological data are still in need for quantitatively predicting the phenomenon and treatment outcomes. For example, the key parameters that the rate of pulmonary cells are killed by viral infection and how fast the apoptosis can attract the immune cells in patients of different ages and physical conditions. In order to capture the dynamics, we although specifically model different kinds of cytokines, we ignore the potential cross-effect of different cytokines, which might be in capable of predicting the treatment effect of different drugs, such as those drugs used for neutralizing cytokines. Moreover, our model did not include the effect of subsequent adaptive immunity. This is because our model only consider the situation where patients get infected with virus for the first time. Future development in models that includes adaptive immunity might give a more complete picture for this disease. Those includes the effect of vaccination and multiple infections on the development and treatment of disease (Liu et al., 2025).

Overall, our mathematical model provides a quantitative framework for disentangling the dynamics of disease progression and its heterogeneity, treatment optimization, especially enabling us to reveal the pathogenesis of COVID-19. Our mathematical framework highlights the importance of deploying the medication at the early stage of disease development. We hope that this framework might be also useful for studying disease progression of other viral diseases both in clinics and animal farms.

## Data Availability

The original contributions presented in the study are included in the article/supplementary material, further inquiries can be directed to the corresponding author.
